# Managing in Critical Care Settings: A Qualitative Study of South African Nurse Unit Managers and the Psychological Contract

**DOI:** 10.1177/23779608231210115

**Published:** 2023-11-07

**Authors:** Linda Ronnie

**Affiliations:** 1 37716 University of Cape Town, Cape Town, Rondebosch, South Africa

**Keywords:** nurse unit managers, psychological contract, critical care unit, employee relationships, global south

## Abstract

**Introduction:**

Little is written about the management of psychological contracts by nurse unit managers (NUMs) in critical care settings and how this perspective contributes to the performance, experiences, and views of nurses and nursing teams. Psychological contracts are important regulators of the employer–employee relationship, with managers (NUMs) being the embodiment of the employer in managing the contract.

**Objectives:**

This qualitative study answers a call for research on the NUM perspective of the psychological contract given the role they play in the wellbeing of critical care nurses and quality of care provided. The study aims to understand the expectations and obligations that constitute the psychological contract NUMs have with their nursing teams, the nature of the contract, and how NUMs practically manage these aspects on their teams.

**Methods:**

Using an interpretive qualitative research design and a purposive sampling technique, semi-structured interviews were conducted with 10 of the 14 NUMs from a public health facility about critical incidents relating to their obligations and expectations of managing critical care nurses.

**Results:**

A thematic analysis of their responses revealed five main themes that represent the contents of their psychological contracts with critical care nurses: professional commitment and obligation; leading by example; trust and support; teamwork; and on-the-job training and further development. In their discussion of these components, the NUMs also revealed how they manage the psychological contract with nurses.

**Conclusion:**

Based on the expectations and obligations NUMs hold with their staff, their psychological contracts were found to be largely relational, with elements of the balanced type, suggesting that they rely on interpersonal connection and coordination, as well as knowledge dissemination, to uphold the contract. This contract appears to be effective in inculcating the commitment of nurses to their profession and professional standards through the building of trust and offering of support. However, recommendations are offered to ensure NUMs are best prepared to sustain these psychological contracts and continue to support nurse wellbeing and related patient care.

## Introduction

Little is written about the management of psychological contracts by nurse unit managers (NUMs) in critical care settings and their perspectives on the obligations they feel towards their job and their nursing team. Critical care nursing describes the care of patients with life-threatening injuries and illnesses. It is complex care often offered with technological assistance by highly skilled clinical practitioners (SANC, 2020), with nursing personnel typically assigned on a nurse/patient ratio of 1:1. Critical care settings have been described by NUMs as stressful places of work with numerous obligations and responsibilities and minimal time available to build meaningful relationships with their teams (Nazari et al., 2016). NUMs report not feeling adequately prepared for their managerial roles (Townsend et al., 2012) and feeling under-valued through a lack of involvement in decision-making (Intas et al., 2021). The work of critical care nursing staff—both nurse and manager—is regarded as intensely emotional with various psychological stressors in a high-risk situation (Mealer et al., 2007). In this environment, where staff face the continual challenges of patient suffering and death, their psychological states can be adversely impacted by these stressors. Prior research has found that behaviors of NUMs—such as establishing trusting relationships, showing progressive leadership styles, support of professional development, acknowledgement, and inclusion in decision-making—positively impact the wellbeing of critical care nurses and thus influence nurses’ ability to provide quality care (Adams et al., 2019a; 2019b).

## Review of Literature

The psychological contract is a construct widely used to examine and understand social exchange relationships in the workplace (Conway & Pekcan, 2019). These exchanges contribute to “an individual's beliefs regarding the terms and conditions of a reciprocal exchange agreement between the focal person and another party” (Rousseau, 1989, p. 123). The concept of the psychological contract was first used to describe the mutual expectations that occur between employer and employees (Argyris, 1960) and is considered implicit (Schein, 1965) as it is influenced by both the beliefs and behaviors of all the parties involved. It is therefore a significant regulator of the employment relationship. Line managers are key to managing the psychological contract as they are a critical source of knowledge, support, and resources for their employees (Baccili, 2001; Janssen & Van Yperen, 2004). The manager, acting as organizational representative, is also ideally positioned to share their expectations and deliver on promises (Herriot & Pemberton, 1997; Rousseau, 2004). Employees are similarly likely to position their manager as the key person for establishing and then maintaining the psychological contract (Shore & Tetrick, 1991). Line managers therefore play a central role in managing the psychological contract that is directly relevant to their team (Petersitzke, 2009). Consequently, the manager's own set of obligations and expectations are a significant lens through which the relationship between their team members and themselves can be viewed.

Line managers have been found to play a significant role in building trust in the relationship between management and nurses in hospitals (McCabe & Sambrook, 2014). Empowerment—through more flexibility over care provision and job flexibility—has also emerged as contributory factor to improved organizational commitment and performance among nurses (Peltier et al., 2013). Positive relationships in the workplace, which are created and shaped by NUMs in the hospital setting, are vital to ensuring that nursing teams are more cohesive, giving nurses a greater sense of value in their work (Laschinger, 2010), and ensuring that patients receive quality care (Purdy et al., 2010). Empowerment and positive relational experiences create more autonomy for nurses, endow their work with deeper meaning, and engender greater job satisfaction.

Although challenges within the healthcare sector, and critical care units in particular, are evident (Christian & Crisp, 2012; Pillay, 2009; Rispel, 2010), it is the duty of managers to foster a work environment where the psychological contract can be fulfilled (Cheung et al., 2016). An authentic leadership style creates greater trust from nurses, which increases their involvement in their job and improves the quality of care for patients (Wong et al., 2010), and also engenders a level of independence in nurses. Stronger trust in leaders also leads to an environment with more open communication and where information sharing is more likely, which can contribute to more effective teamwork (Dietz et al., 2014) and reduce the likelihood of clinical error (Goel & Yang, 2015).

Leaders are responsible for modeling the values and ethos of the profession in order to inculcate them in employees (Tanaka et al., 2016). In the nursing context, where there is a large relational component to the work and a strong emphasis on professional values, modeling and practicing of these values provide important indicators of the psychological contracts of nurses and by extension, NUMs. The relationship between critical care nurses and NUMs as their line managers is therefore instrumental in shaping the psychological contract as direct supervisors come to “personify” the employer in the eyes of employees (Suazo et al., 2009).

Pertinent to the management of the psychological contract is its type, of which there are three: transactional, relational, and balanced (Robinson et al., 1994; Rousseau, 1995). While transactional contracts tend to be unambiguous in their mutual expectations around a prescriptive set of duties and require less loyalty to the particular organization of individuals, relational contracts indicate a deeper commitment between parties related to the responsibilities of the job and goals within the organization. Balanced contracts take a more dynamic view of employee and employer interface, with parties seeing opportunities to advance the performance of the individual (both at the organization and with future employers) and the organization (both in current duties and increasingly more demanding duties), over time. As Bunderson (2001) outlined, the psychological contracts of professionals are likely to be relational as the contract should contain elements such as provision of a collegial work environment, upholding of professional autonomy and standards, and sense of identification, loyalty, and fulfillment of the obligations of the role. However, this is contrasted by the more transactional nature of the role, i.e. the administrative element or exchange ideology where the focus is on fulfilling more formal obligations, such as working within hospital budgetary frameworks and completing administrative tasks such as resource planning. This “tension” may reflect in the perspectives of the NUMs in this study as nurse managers—like supervisors in other settings—hold a position where the complexity of the role means being responsible for implementing (in their case) hospital policies and processes while shaping role expectations and monitoring the performance of their staff.

### Objective of the Study

This study sought to investigate the obligations and expectations implicit in the psychological contracts of NUMs and their nursing teams, the nature of the contract, and how NUMs manage these aspects on their teams. The research answers the call by Adams et al. (2019b) for further studies on NUM perspectives to explore the supportive role that these managers can play in reducing critical care nurse burnout and improving wellbeing, as these may in turn reduce nurse attrition and improve patient outcomes. The psychological contract therefore provides an ideal framework for examining the management practices of NUMs and how they may or may not support the wellbeing of critical care nurses and quality of care for patients.

## Methods

### Design

The study took an interpretative, qualitative approach. Interpretative methodologies aim to understand the significance and intentions of people's actions and interactions based on their own descriptions of those events and experiences (Elliot & Timulak, 2021). Using a purposive sampling approach, nurse managers in a critical care setting at a local tertiary hospital were selected to participate in the study. The experiences of nurse managers were sought as the manager–nurse relationship is known to be significant in creating beneficial psychological contracts that influence healthcare delivery, patient care outcomes, and lower turnover (McCabe & Sambrook, 2014). It is further argued that a context where decisions have the potential to impact patient survival allows for a far richer and deeper mapping of managers’ views of the psychological contract.

### Research Questions

Interviews with NUMs were used to answer the following research questions:

*RQ1: What obligations and expectations do Nursing Unit Managers have for their nursing teams?*
*RQ2: How do they manage those obligations and expectations on their teams?*

### Sample

The setting for this study was a public, tertiary hospital in the Western Cape, South Africa, with critical care units comprising neuro-surgical, trauma, cardio-thoracic, and patients with high anesthetic or respiratory risk. The full complement of 14 nursing unit managers meeting the selection criteria in the critical care units of this hospital was contacted to participate in the study, with one declining due to workload and three due to concerns regarding confidentiality. The final sample consisted of 10 nursing unit managers across the four critical care units that were similar in size and staffing. Information about participants’ departments was not collected to ensure confidentiality. As Table 1 shows, the participants’ nursing experience ranged between 10 and over 35 years with their management exposure ranging from less than 1 to over 20 years’ experience as a critical care NUM. Beyond their on-the-job training and nursing experience, no participant had received any formal management training. All had been recruited for their NUM roles from the pool of Registered Nurses on the critical care units and held a formal, accredited qualification in the critical care field.^1^

**Table 1.** NUM Participants: Gender, Years of Nursing Experience, and Management Experience.^2^

Name	Gender	Nursing experience (years)	Management experience (years)
Alex	Male	>25 and <30	>1 and <2
Serena	Female	>30 and <35	>10 and <15
Delia	Female	>20 and <25	>15 and <20
Buhle	Female	>15 and <20	>3 and <4
Grace	Female	>35	>10 and <15
Pule	Male	>10 and <15	>0 and <1
Lena	Female	>35	>3 and <4
Susie	Female	>35	>10 and <15
Thuli	Female	>20 and <25	>4 and <5
Cathy	Female	>25 and <30	>20

### Inclusion/Exclusion Criteria

All nurse managers employed in the critical care units and who were full-time employees at the hospital site were included in the study. The research excluded those who were temporary NUMs or held managerial roles in traditional wards.

### Ethical Considerations

Ethical clearance to conduct this research was obtained from the relevant university. The participation of NUMs was initially negotiated directly with the hospital. In a subsequent meeting facilitated through the Head of Critical Care, the purpose of the study was explained, confidentiality was highlighted, and NUMs assured that they were under no obligation to participate. All participants provided verbal recorded consent at the start of their interview when the voluntary nature to participation was reiterated. Participant names have been anonymised and some identifying information removed in the reporting of the data to ensure privacy of the individuals and organization.

### Data Collection

Interviews were conducted on a once-off basis to ensure minimal disruption to work. The interviews took place in a quiet private setting away from the critical care wards in late February 2020. The interview schedule consisted of open-ended questions. Following the critical incident technique (Flanagan, 1954, cited in Bryman & Bell, 2018), managers were asked about specific situations or experiences that they found significant (“critical”) while managing and supporting their nursing staff. The average recorded interview—later transcribed—took 28 min and ranged between 21 and 42 min. The in-person interviews consisted of two segments: (a) demographic questions about the NUMs overall nursing and critical care management experience, and their qualifications, and (b) semi-structured questions regarding their role and relationships with their nursing staff in their critical care managerial role. To give them the opportunity to relate their story, each nurse manager was asked: “What have been your experiences of managing in your unit?”. To obtain more detail, follow-up prompt questions were used: “Provide an example of that” or “As a nurse manager, how did that make you feel?”. In addition to recording the interviews for later analysis, the interviewer took handwritten notes to capture keywords and descriptions in real time.

For a qualitative interpretive study, the number of interviews needed for data reliability is determined by the saturation point in the interview schedule, which is reached once the information and descriptions from participants become redundant (Trotter, 2012). Although 10 interviews were scheduled, a reduction in new ideas was observed after eight interviews and data saturation was reached after the ninth interview. The 10th interview was therefore used for additional verification purposes.

### Data Analysis and Trustworthiness

The critical incidents from the interviews were analyzed using an inductive thematic analysis. Following the methodology by Braun and Clarke (2006), the analysis involved the researcher reviewing her interview notes, familiarizing herself with the data, generating initial codes, searching for themes in the incidents, reviewing the themes, and defining the themes. The thematic analysis was conducted by the researcher, while two colleagues supported data validation by reviewing and checking the interpretations of the researcher. During the code generation, theme identification, and review processes, exact phrases and words of participants were extracted from their descriptions of “critical” experiences for use in the findings and analysis. The researcher reviewed these incidents for common themes or recurring phrases, sentiments, ideas, and concepts. An iterative coding procedure was used to confirm the meaning of the extracted excerpts. Trustworthiness and authenticity of the research process were essential. To this end, best practices for qualitative research were followed to establish credibility (Spencer et al., 2014). Dependability was supported through careful recording of interviews via digital means and note-taking, and through safeguarding of data and associated records. The reliability and credibility of the data analysis were reinforced through the practice of peer debriefing with two colleagues as a form of “member checking” (Bazeley, 2013). The peer debriefing process also mitigated any risk of researcher bias in interpreting the interview responses as it enabled alternative perspectives to be voiced and, where appropriate, for further clarifications and changes to be made (Leedy & Ormrod, 2019).

## Findings

NUMs in critical care units, which provide treatment for patients with life-threatening injuries and illnesses, are responsible for managing critical care nurses, as well as providing care themselves. Management duties include scheduling nurse shifts and facilitating teamwork within the unit; training and mentoring nurses, and assisting with their career development; assuring quality of care provided by nurses; and overseeing the safety and hygiene of the work and patient care environment. In their capacity as nurses, NUMs work alongside their teams to treat patients, clean equipment, and perform other tasks necessary to the care of patients.

The critical incidents that NUM participants described in terms of their work obligations and expectations focused on five main themes: professional commitment and obligation; leading by example; trust and support; teamwork; and on-the-job training and further development. Certain participant experiences often reflected an overlap of multiple themes, demonstrating the ways the elements of the psychological contract are interconnected for NUMs.

### Professional Commitment and Obligation

All NUMs expressed overwhelming commitment to their critical care patients. It appeared that, even though the NUMs had moved into managerial roles, their nurse identity remained an integral part of who they were. As Lena explained: “I am here to do a job and, because I’m appointed to do that job, this is what I want to do, and this is how I work. It's not for me, it's for the patient.” Serena concurred, saying that her overarching role was, “to observe safety for the patient – to create a safe environment for the patient and quality care for the patient”. Buhle's view was that in a critical care setting you were dealing with the most vulnerable of all: “I’m always an advocate for the patients. That is one thing since my training that I’ve learned, you have to be the spokesperson for your patients because the patients on the ventilator they can’t speak for themselves. Nurses must treat a patient the way they’d want to be treated.”

Pule noted how he gave his nursing team the utmost respect, “but when it comes to work, I can’t be seen to be doing the wrong thing. I understand that we have different ways of doing things. And as long as we serve our patients, then I’m okay with it, as long as what [the nurses] are doing won’t come back to us or won’t harm the patient.” Delia provided an example of how poor work standards would have an effect on everyone, not just on a specific unit. She described what she had found when she had come on duty earlier that evening:

“The patient's endotracheal tube was so dirty. I said to the team, ‘Before I do anything else let's change the tapes and redo the tube.’ Because if this person has a visitor now, that is going to reflect on all of us as nurses because people are going to say the nurses of [this hospital], they’re not going to name a specific nurse that was allocated to the patient (Delia).

Other NUMs echoed this sentiment through constantly reminding their teams that their work must be kept up to standard so that each nurse could, at the end of the day, leave the bedside, knowing the patient was safe and that they had done their best. This commitment and interest in the patient extended outside of the critical care unit. Thuli described her feelings regarding a patient's recovery:

“The thing I like most about my job is when your patient is getting out of ICU to the ward, and they come back to say thank you. The family sometimes brings them in to show us how they look and that they’re walking. That is something that really steals your heart” (Thuli).

Susie shared the progress of a patient who has been in the critical care unit for 96 days.

“On a daily basis, when [I] come into the unit and see him still alive, it was so encouraging. And the other day when he walked in here, just before he went home, everybody was so excited to see him. We felt we had been part of the success – using our experience and our knowledge” (Susie).

### Leading by Example

NUMs conveyed a clear and uniform understanding of their professional obligations in terms of their units. With regard to their staff, they felt they needed to lead by example. As Cathy noted:

“I must be a role model. There should be good communication. You must know all the patients. You must see that your stock is here, the place is clean and a safe environment to work in. If you interact with your staff and work with them together, hand in hand, they will respect you and your unit will be run smoothly” (Cathy).

This view was echoed by Serena: “You must set a good example. You can’t expect people to do a sterile procedure and then you do it in an unsterile way. You must set high standards for the unit as far as possible.” Susie felt that a good NUM should work with their staff: “To be in the struggle with your staff and not to manage from your office, but to manage with them and to give them that space to grow, allow them their opinions and for me to accept criticism.” Alex noted that if his team had worked with him for a few months, they would “know exactly what you want, what you like for your patients, how you’re going to do things and they will catch up and they will keep that standard. You can trust them because that is how you trained them.” Delia also described visibility as a key factor: “You should have an open door policy so you must be visible in your area. You can’t be behind closed doors, and not know how your staff is doing inside in the unit. You have to show interest.” Grace provided an example of role modeling in respect of sanitizing medical equipment.

“We have to keep the equipment clean, but nurses feel it should be the cleaners’ work. The cleaners don’t want to do the equipment because they’re scared that they’re going to break it. So, we’re at the stage now where nobody wants to do anything. Then I said ‘okay, I’ll do it’. Hopefully by doing those kinds of things, you can get them on board” (Grace).

### Trust and Support

Trust between nursing staff is essential in a critical care setting. NUMs have various ways of showing their trust in their staff or building a trusting environment. Lena shared her method: “I leave the unit so that they can see that I do trust them. But I don’t know if they see that for what it is. However, I know that they will come and ask me if there is something that they need to be done, then I will go inside and assist.” Cathy preferred to watch from a distance and then “go and check because the moment you show you don’t trust a person or you are on top of that person, things can go badly wrong because they’re stressed, or they’re worried because you’re on their tail all the time.” A good nurse manager, in Buhle's opinion, was “somebody that can listen. Somebody that can trust the staff. Somebody that can recognize that there are different personalities and support them appropriately”. In terms of providing support, Susie explained her view:

“I’m here to support my nurses too and for me, although I’m a manager, I’m still a nurse. I’m still part and parcel of the workforce and I always do all my admin work in the unit. The only time when I use my office is when I interview somebody or when I do something on the computer, but most of the time, I’m in the unit and I support my staff. They know they can come to me whenever they want to” (Susie).

Further examples of support were expressed by Serena and Delia. However, these were statements of professional support, i.e. support that strictly pertained to medical care and professional duties in the work environment.

“Communication is very important. When you listen, you will find a problem area that needs attention. You can see if you can do something. But I can’t be a people pleaser also, hey? I will have to stick to the regulations of the hospital – staying within the regulations of the hospital at all times, but where possible, I help” (Serena).

“You have to be sympathetic with your staff and you have to give them space. Ask them if anything is bothering them or if they feel okay. Sometimes you also have to be strict especially if they want to take shortcuts. Then you have to be strict and say, ‘Listen you don’t, you can’t, take shortcuts in nursing’. It's either you’re doing the right way, or you ask somebody for help” (Delia).

“There are never arguments because if somebody's doing something wrong, we have a moment where we discuss, and I will tell them this was something I didn’t like, where procedures weren’t followed. It's important to say things to people when they’re around. That is how I build my relationship with the staff, and I also praise them. If, like last night we worked hard, this morning I will say to them, ‘Thank you all because although it was a tough night we pulled through’” (Thuli).

### Teamwork

In terms of teamwork, there was a clear sense that everyone in the unit is in the situation together. The term “family” was used by six of the participants. Some examples of how this might be seen in the critical care units were as follows:

“We’re working very well as a team; it's like a family. We’re more at work than we’re at home and we try to understand each other or know each other's moods. We know each other's shortfalls, help each other along, and see that the work gets done with no negative outcome” (Cathy).

“It's not like I’m the manager, you work under me. We all are one. We all work as one” (Buhle).

“We work as a family. We are colleagues. We know when to support each other and we know, listen, leave it for today. The staff in the unit is very motivated. They are really hard working. I always say to them, ‘See [the unit] as the kingdom. Because this is where we spend most of our time and we have to give our best and we have to support each other’” (Susie).

Serena strongly felt that “if you’ve got a good healthy team spirit and a healthy environment, then they are all happy—they want to help. Somebody offers their help and says, ‘Is this supposed to be done? I’m available. I’ll be prepared to help you there’. It's beneficial for everybody in the unit.” For Buhle, admitting new patients was a frantic time “When there's a lot of work, then we team together up. When the unit is very, very busy, we’re not getting on top of things, then we just help one another with everything. ‘Is your part done? Okay, not done. What can I do for you?’ And so that's how we do it.” The reality of the critical care context means that there is a lot of sustained pressure but “at the end of that pressure, everyone here is honest. They will tell you, ‘This is not right. We should fix that’ in a positive way. All of us, we know what's expected of us and we try by all means to live up to that” (Pule).

### On-the-job Training and Further Development

As the research context was a teaching hospital, the theme of providing training to nurses was quite prominent. As Serena explained:

“Every moment is a training moment or opportunity. So, you’ll make use of all the opportunities to train your staff, encouraging them to go on courses. There are a lot of opportunities which you can give them. And if there are those who are a little bit reluctant, you can encourage them to go” (Serena).

The notion of sharing knowledge is critical to the nursing profession and more so within the critical care unit. Alex felt strongly about knowledge sharing and that his role was as follows:

“To communicate with everybody [so that they] feel free to ask any question. If the person is unsure about something, don’t do it. Come to me, I will show you how to do it. It doesn’t matter how many times I am going to show you because this is life. What if it's your mother, your father, your sister, your brother?” (Alex).

For Cathy, it was about building the leadership pipeline as well. She explained that she would assign specific nurses to run the shift:

“Because everybody must learn. Tomorrow if someone is not here or sick and then at least that person knows what to do. I think that also, they see that trust when I say, ‘You take this shift today, it's your turn’. It's sharing that responsibility and training them up” (Cathy).

This was echoed by Pule: “I allow them to mentor and train others, for example the student nurses. It makes them feel important and also gives them a sense of belonging so they can feel ‘there's a space for me in the unit. I am appreciated’.”

Not all on-the-job training proceeds smoothly and NUMs have to be vigilant and observant at all times. As Serena explained: “If you delegated a task, you would have to go and check if it is done because quite often you will find something isn’t done properly. That is part of delegation. I can’t leave it at that because it's a risk in terms of the patient's life.” Delia agreed:

“Part of my responsibility is to sort out problems especially if the staff nurses or junior nurses are allocated to a patient. You have to make sure that they know what they’re doing around the patient's bed. You have to be observant and see to the need of the patient, otherwise proper patient care won’t be given” (Delia).

Susie had been a micro manager in the past and explained:

“If you were slow, I would take things out of your hands. But I’ve said to myself – I’m actually doing harm to that person. Now I will monitor them and if I see, this is moving in a direction where a disaster is going to happen, I’ll jump in, but I will let people take the initiative and do things for themselves” (Susie).

As Serena had alluded to earlier, further education is available to less experienced nurses. In Buhle's case, she said to one of her junior nurses, “‘When the colleges are open, go for further education. You can be a Sister, a good Sister. I feel when you get there, you won’t struggle because most of the things you were helping us with’.” This was reiterated by Susie, who also supported nurses to further their studies:

“I encourage and help the nurses here with their studies so that they can improve themselves to be registered nurses, ICU trained. That makes me proud. There are opportunities and people don’t grab it. I will help – even if I have to come in over a weekend when we can discuss” (Susie).

## Discussion

Professional identity is at the heart of the obligations and expectations NUMs hold for their nursing team. Their insistence on upholding high-professional standards, which they expressed repeatedly when discussing all aspects of their work, may be particularly acute given their location in the critical care units where patients are especially vulnerable. The concept of professional commitment has been described as a psychological state that triggers employees to maintain their membership of a professional group (Jourdain & Chênevert, 2010; Meyer & Herscovitch, 2001). According to Meyer and Allen's framework (1991), the psychological commitment of individuals is based on their emotional attachment to their profession (Blau, 2003; Lee et al., 2000). As can be seen in the NUMs’ experiences, not only do they exhibit loyalty to the profession, but they attempt to inculcate that in their interactions with their team through role modeling and setting an example. This is encouraging as prior research found links between nurses’ professional commitment and the motivation of teams (Galletta et al., 2019), an intention to improve and upgrade professional competence and capability (Chang et al., 2021), job satisfaction (Carcati et al., 2014), and patient care (Teng et al., 2019). The focus of the NUMs on the provision of quality healthcare and ensuring professional care of critical care patients is a central element of a relational type of psychological contract (Jones & Sambrook, 2010). Through leading by example, NUMs in this study played a critical role in creating an inclusive culture that sought to promote a healthy psychological contract. The expressions of interest, being visible, and role modeling show further elements of a relational contract (Rousseau, 1989).

Nurses expect NUMs to demonstrate trust and confidence in their abilities and to support them by granting them a level of autonomy. In this study, much of this autonomy was implicitly exhibited, as in the case of Lena and Cathy who both showed trust through their absence from the unit. NUMs’ primary focus on their professional commitment meant that their support to their staff was not offered value-free. The nature of work context with its critically ill patients means that critical care units are among the most stressful and demanding environments (Jakimowicz et al., 2018). Consequently, the context—and its attendant overall accountability and responsibility for the NUM—influences the level of trust that can be shown between individual nurses and their managers, especially in light of evidence that while they are registered nurses, only 25% of the nurses in intensive care hold the requisite qualifications to be there (De Beer et al., 2011).

Teamwork is therefore a crucial aspect in managing the critical care unit to ensure everyone is able to carry out their responsibilities effectively. Furthermore, the notion of “family” creates an organizational dynamic and culture conducive to improved morale and is pivotal in promoting the relational aspects of the psychological contract (Mallette, 2011). A teamwork ethos is also indispensable because patient care may be compromised when workplaces are non-supportive and non-collegial (Laschinger, 2010). Through this building of the collective, encouragement of team support, and relational interdependency, NUMs inadvertently also contribute to building peer-to-peer psychological contracts which have been found to be present amongst critical care nurses, resulting in greater trust and communication between nurses. In a critical care context, collaboration is even more essential as a lack of mutual regard can contribute to daily stressors (Siffleet et al., 2015).

NUMs in this study showed support for both on-the-job training and further development forms. While the context—that of a teaching hospital—created a conducive climate, it was clear that NUMs were encouraging towards their staff and comfortable with sharing their expertise. Studies confirm that critical care nurses need appropriate training to deliver relevant and professional care (De Beer et al., 2011) and indeed request this training themselves. In terms of managing risk with workplace training, NUMs have to be vigilant but are keen to move away from micro-management in this regard. Formalized training in the form of critical care qualifications are provided by nurse colleges and NUMs do their utmost to encourage their staff to attend, supporting them through this training in their own time. Succession planning is also important and examples of building the leadership pipeline are evident. These two avenues—professional training and succession planning—can be understood as crucial to NUMs in this study, all of whom have had little, if any, managerial training. Past studies have found that NUMs often move to more administrative roles with minimal skills and knowledge around people management (Baxter & Warsawsky, 2014). The lack of preparedness means that NUMs learn how to manage staff—and consequently, their psychological contracts—through trial and error. This lack of insight can cause frustration and a lack of self-efficacy (McCallin & Frankson, 2010).

## Strengths and Limitations

This study addressed a gap in the literature regarding the management of psychological contracts by NUMs in critical care settings and how this perspective contributes to the performance, experiences, and views of nurses and nursing teams. Limitations of the study include that it presents only the perspectives of critical care NUMs which may differ to those NUMs in other contexts. Furthermore, the study only draws on a single hospital in one geographic area. Both of these shortcomings present opportunities for further research as does the exploration of the central role of the NUM in a critical care setting. Another possible avenue of study is around the psychological contract between NUMs and their managers to explore how fulfillment or breach between the parties impacts the relationship between NUMs and their staff. Given the importance of professional commitment, investigating the psychological contract between NUMs and patients may also prove fruitful.

## Implications for Practice

NUMs have a significant role to play in managing the psychological contract of and inculcating commitment from critical care nurses. As critical care settings are high-performance, high-stakes environments, a few suggestions for improvement may be pertinent. The psychological contract needs to be made explicit through NUMs sharing their expectations. Open communication can ensure that critical care nurses’ psychological contracts are fulfilled and remain positive. As the NUM creates a culture of respect and support, an ongoing commitment to be present and supportive is needed. Given much of the intent appears in place, it is simply a matter of reinforcement. However, leadership and management training in inter-personal skills, communication, and negotiation may address any possible areas of improvement and will actively support succession planning for those nurses prior to managerial appointments.

With regards to encouraging further staff development, NUMs are to be congratulated for their efforts in this regard. However, appropriate staffing—both in numbers and in skill—of the critical care units by senior management will go some way towards easing the developmental burden on the NUM. NUMs should build on their existing notion of family and ensure team relations and spirit remain conducive as peer-to-peer psychological contracts between critical care nurses are key, both for creating collegial environments but also for improving patient care.

## Conclusion

This study found that the expectations and obligations of the psychological contract NUMs have with their nursing teams' center on five key elements: professional commitment and obligation; leading by example; trust and support; teamwork; and on-the-job training and further development. These contents are related to various employee behaviors where a useful interpretation is that of social exchange. In this case, NUMs manage these obligations and expectations on their teams through seeking to build a sound psychological contract and signaling key aspects of importance, expecting reciprocity from their nurses. The findings therefore appear to indicate that a predominantly relational contract (through trust, support, encouragement, and teamwork) with few elements of the balanced type (provision of training and development, the creation of a safe working environment, and professional commitment) are present. This psychological contract, and the management practices that maintain it, appears to promote nurse wellbeing and quality of care for patients and therefore may provide an important framework for nurse managers. The professional commitment of NUMs is commendable. Retaining their nurse identity, and the commitment that implies, allows NUMs to understand the challenges that their critical care nurses experience daily and, as such, bridge the gaps in the employee–employer interface.

## Notes

1. For further explanation, see https://www.sanc.co.za/wp-content/uploads/2020/06/SANC-Competencies-Critical-Care-Nurse-Specialist-Adult.pdf

2. Pseudonyms have been used for confidentiality purposes.
